# [Corrigendum] lncRNA TPTEP1 inhibits stemness and radioresistance of glioma through miR-106a-5p-mediated P38 MAPK signaling

**DOI:** 10.3892/mmr.2025.13605

**Published:** 2025-07-01

**Authors:** Ting Tang, Ling-Xing Wang, Mei-Li Yang, Rong-Mou Zhang

Mol Med Rep 22: 4857–4867, 2020; DOI: 10.3892/mmr.2020.11542

Subsequently to the publication of this paper, an interested reader drew to the authors’ attention that the control β-actin western blots shown in Fig. 4D on p. 4864 were strikingly similar to control blots that had appeared in another paper by the same group published previously in the journal *Biochemical and Biophysical Research Communications*.

The authors have re-examined their original data, and realize that this figure was assembled incorrectly. The revised version of Fig. 4, now featuring the correct β-actin western blots in Fig. 4D, is shown on the next page. The authors sincerely apologize for the error introduced during the preparation of this figure, although they confirm that this did not grossly affect either the results or the conclusions reported in this study. They also thank the Editor of *Molecular Medicine Reports* for allowing them the opportunity to publish a Corrigendum, and regret any inconvenience caused to the readership.

## Figures and Tables

**Figure 5.**
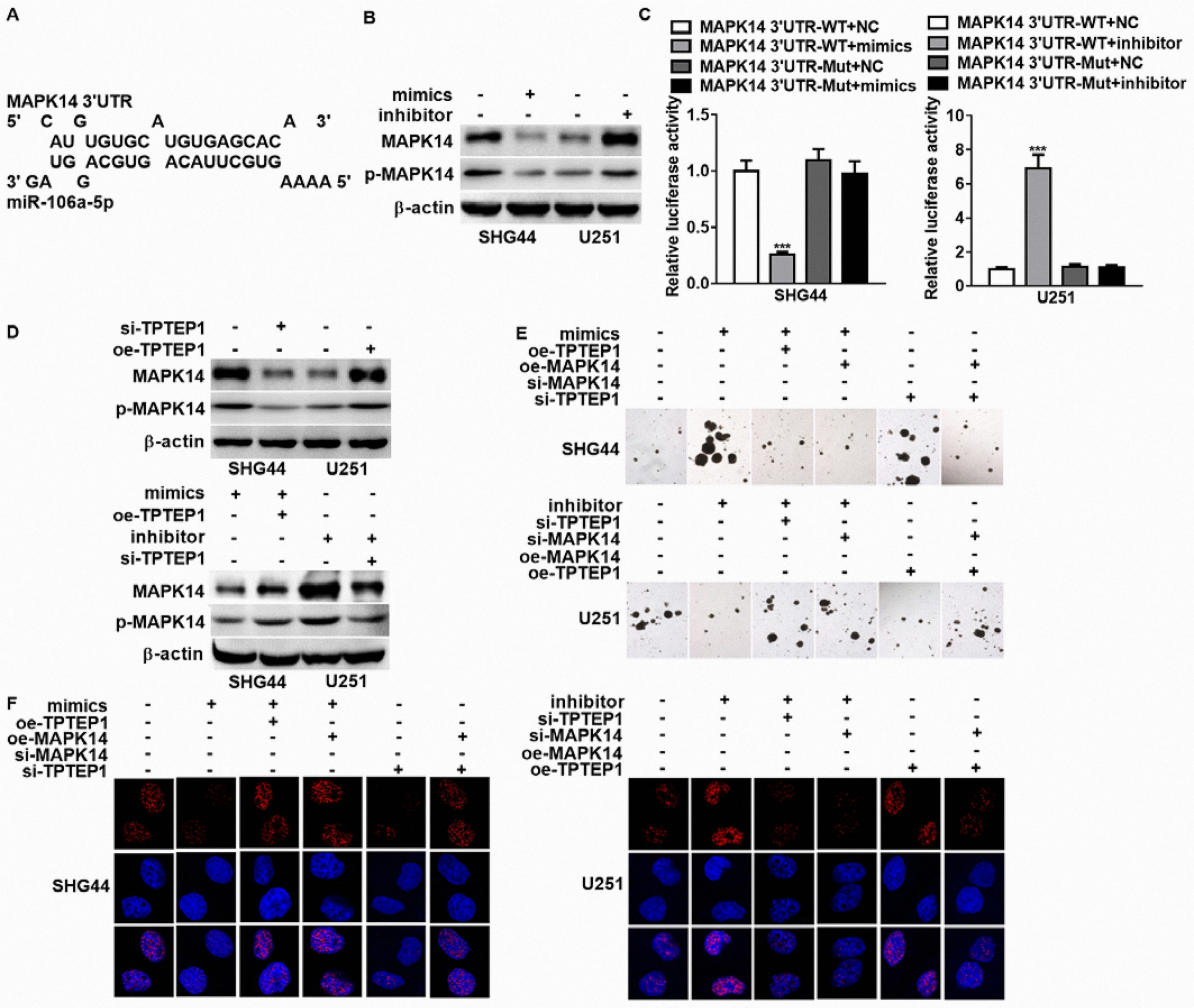
The TPTEP1/miR-106a-5p/MAPK14 axis in glioma. (A) *In situ* hybridization analysis of TPTEP1 and miR-106a-5p, and immunohistochemical staining for MAPK14 in glioma. Correlations among TPTEP1, miR-106a-5p and MAPK14 expression levels (representative images) (Spearman's rank correlation test). (B) Working model of TPTEP1/miR-106a-5p/MAPK14 axis in glioma. TPTEP1, transmembrane phosphatase with tensin homology pseudogene 1; miR, microRNA.

